# A novel spraying nanoprobe for renal cell carcinoma in humans

**DOI:** 10.1093/lifemedi/lnac059

**Published:** 2022-12-23

**Authors:** Qi Chen, Lu Chen, Yushan Liu, Wenzhi Li, Qing Zhong, Bin Xu, Zhong Wang, Weiwei Wang

**Affiliations:** Department of Urology, Shanghai Ninth People’s Hospital, Shanghai Jiao Tong University School of Medicine, Shanghai 200011, China; Department of Urology, Ruijin Hospital, Shanghai JiaoTong University School of Medicine, Shanghai 200025, China; Department of Urology, Shanghai Ninth People’s Hospital, Shanghai Jiao Tong University School of Medicine, Shanghai 200011, China; Department of Urology, Shanghai Ninth People’s Hospital, Shanghai Jiao Tong University School of Medicine, Shanghai 200011, China; Key Laboratory of Cell Differentiation and Apoptosis of the Chinese Ministry of Education, Department of Pathophysiology, Shanghai Jiao Tong University School of Medicine, Shanghai 200025, China; Department of Urology, Shanghai Ninth People’s Hospital, Shanghai Jiao Tong University School of Medicine, Shanghai 200011, China; Department of Urology, Shanghai Ninth People’s Hospital, Shanghai Jiao Tong University School of Medicine, Shanghai 200011, China; Key Laboratory of Cell Differentiation and Apoptosis of the Chinese Ministry of Education, Department of Pathophysiology, Shanghai Jiao Tong University School of Medicine, Shanghai 200025, China; Ningbo Haibo Biotechnology Co., Ltd, Ningbo 315615, China

## Dear editor,

Nephron-sparing surgery (NSS) is one of the most important procedures for renal cell carcinoma patients with a purpose to minimize the risk of metastasis yet preserve kidney function [1]. However, positive surgical margins (PSMs) are sometimes inevitable following NSS and are associated with higher rates of local recurrence and worse overall survival [2, 3]. Rapid reacting and non-toxic tumor-specific contrast agent is lacking in clinical settings to detect PSMs in a timely manner [4, 5]. Here, a nanoparticle was designed, synthesized, assembled, and applied as a tumor-specific contrast agent. We showed that it could identify the surgical margins in a few minutes in clinical application. In addition, different from traditional contrast agents, our nanoparticle could be directly applied to renal tumor tissues by spraying, without the need for an intravenous injection.

**Figure 1.**
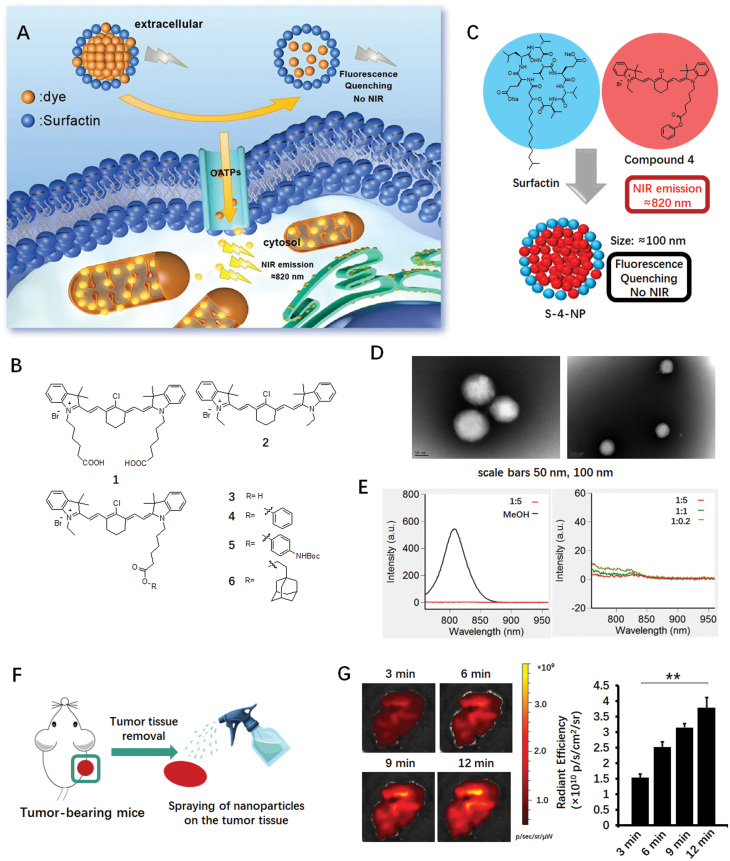
**Design and functional validation of the near-infrared spraying nano particles in detecting tumor boundaries in mouse tumor models.**(A) Schematic illustrations showing the active release mode of the spraying nanoparticles. OATPs, organic anion-transporting polypeptides; NIR, near-infrared. (B) Chemical structures of compound **1-6**. (C)The illustrated structure of compound 4-based nanoparticles, named **S-4-NP**. (D) Transmission electron microscopy images of compound **4** combined with surfactin in a 1:5 molar ratio. Scale bars: 50, 100 nm. NIR, near-infrared. (E) Fluorescence spectra of compound **4** combined with surfactin at different molar ratios (excitation at 730 nm; emission from 750 to 960 nm). (F) Overview of the xenograft animal model experiment. (G) NIR imaging study of ompound **4** applied to 786-0 tumor-bearing mice (*n* = 3) (representative fluorescence images, left; quantitative analysis results, right). **P < 0.01.

A schematic diagram of our study is illustrated in Fig. 1A. A near-infrared (NIR) hypoxia-targeting dye (**dye**) [6] and an anionic surfactant (**surfactin**) were self-assembled into nanoparticles in aqueous solution [7]. The fluorescence of the dye was self-quenched due to close proximity as a result of the particles’ nanostructure. When the nanoparticles came into contact with cancer cells, the dye specifically recognized the organic anion-transporting polypeptides (OATPs) on the cell membrane, was released from the nanoparticle structure, and accurately entered the cell. Inside the cell, the fluorescence of the free dye was restored, thus marking cancer cells with a NIR signal. Based on this mechanism, our nanoparticle could be directly sprayed onto the surface of cancerous tissues to enable real-time tumor imaging.

A series of heptamethine indocyanine dyes (compound **1**-**6**), with a rigid 1-chlorocyclohexenyl substitution in the middle of the polymethine linker, were designed and synthesized (Fig. 1B). Recent reports suggested that these specific chemical structures could enable dyes to signal hypoxia-dependent uptake by cancer cells through activation of the HIF1α/OATPs signaling axis and to further accumulate in the mitochondria [8]. Human 786-0 cells were used to assess the cellular uptake and subcellular localization of the dyes *in vitro* (Fig. S1).

The nanoparticles were generated via the self-assembly of dyes and surfactin in aqueous media as shown in Fig. 1C. We characterized the sizes of the nanoparticles from different dye and surfactin combinations by both dynamic light scattering (Table S1) and transmission electron microscope (Figs. 1D and S2). The results demonstrated that particles from compound **4** had the maximum hydrodynamic diameters (around 100 nm), while particles from compounds **5** and **6** had the minimum diameters (around 50 nm). However, we could not detect nanoparticles from compound **1**, **2**, or **3**.

We next examined the quenching effect of the dye fluorescence by surfactin by keeping the concentration of the dyes at 20 μM while changing dye-to-surfactin molar ratios from 1:5, 1:1 to 1:0.2 (Fig. S3). For all the dyes examined, incorporation of surfactin dramatically reduced the fluorescence intensity compared with that of the free dye in methanol solution. The quenching effect was the most pronounced for compound **4**, **5**, and **6**, and the fluorescence almost disappeared at a dye-to-surfactin molar ratio of 1:5. The fluorescence spectra further demonstrated the quenching effect. As shown for compound **4**, the emission peak (at 810 nm) completely disappeared compared to the methanol solution (Fig. 1E). Similar results were observed for compound **5** and **6** (Fig. S4). Different from the complete quenching effect observed for compound **4**, **5**, and **6**, the quenching effect was incomplete for compound **1**, **2**, and **3** (Fig. S4).

Combining the morphology results and the fluorescence quenching results, we conclude here: (i) the high local concentration of the dye in the particles quenched the fluorescence, which is a classical self-quenching effect [9]; (ii) for compound **1**, **2**, and **3**, the interaction between surfactin and the dye was weak so that some free dye remained in the solution, resulting in incomplete quenching; (iii) for compound **4**, **5**, and **6**, the interaction was strong enough that nearly no free dye was left in the solution, resulting in a stable system with complete quenching of fluorescence. These results indicated that the large organic moieties in the molecular structures of compound **4**, **5**, and **6** effectively increased the stability of the particles.

The 786-0 xenograft mouse model was used to evaluate the tumor-targeting properties and NIR imaging profiles of our nanoparticles (Fig. 1F). Tumors were first removed from the xenograft model from the mice, the solution with compounds was then immediately sprayed onto the cancerous tissue. NIR images were obtained at 3, 6, 9, and 12 min after spraying (Figs. 1G and S5). For compound **1** and **3**, a strong uptake value was observed at 3 min, after which the signal intensity no longer increased. This result suggested that those particles were easily dissembled and the self-quenching effect disappeared when they came into contact with the biological tissue, due to the unstable microstructure. In contrast, for compound **4**, **5**, and **6**, there was an obvious increase in the uptake value (>2-fold) from 3 to 12 min, indicating a higher stability of these nanoparticles, consistent with our *in vitro* data. To assess the hypoxia-targeting properties of these compounds, we used a reported OATP1B3 selective inhibitor (bromosulfophthalein) as a competitive inhibitor of compound **4**. At a oversaturating dose (50 times eq.), bromosulfophthalein significantly decreased the uptake value of compound **4** by ~85% (Fig. S5F).

Most of the previously reported nanoparticles enter the target cell as a complete structure, releasing the loaded drug intracellularly. In contrast with these traditional drug delivery systems, our system featured an active release mode: only loaded dyes entered the targeted cell, rather than the entire nanoparticle. To verify this unconventional release mode, we designed experiments. The results and discussion were showed in Fig. S6.

**Figure 2.**
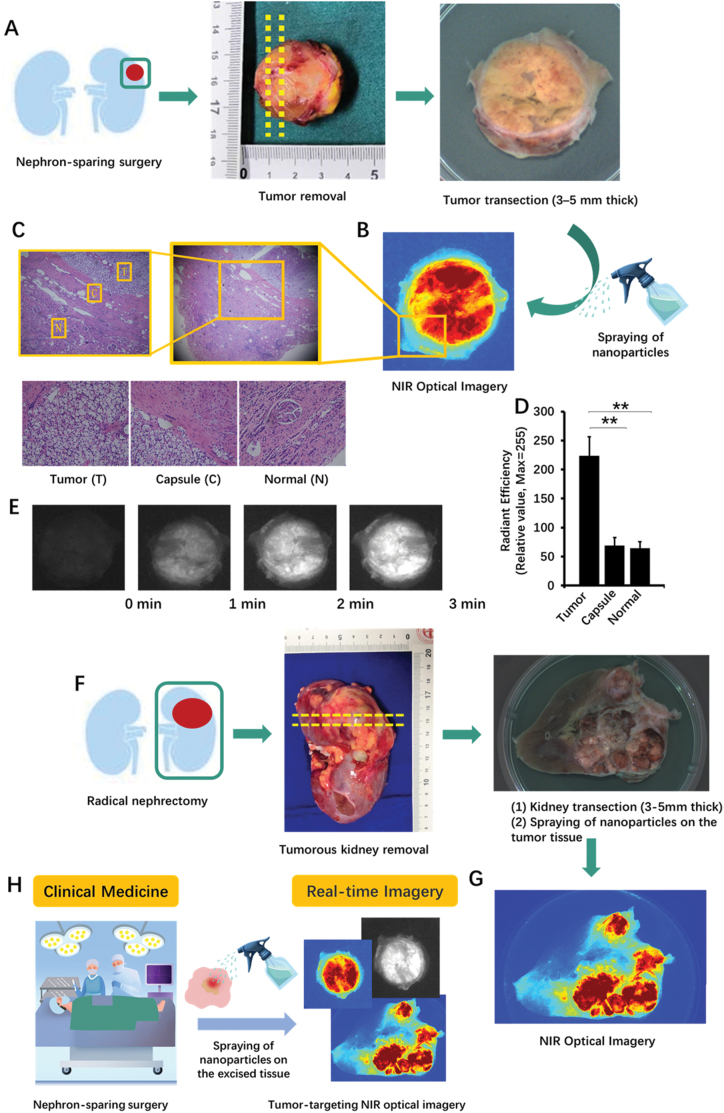
**Validation of the near-infrared spraying nano particles in detecting tumor boundaries in human kidney tumor samples.**(A) llustration of **S-4-NP** applied in a NSS. (B and C) Representative pseudocolor image and hematoxylin/eosin staining result of the transected tumor sprayed with **S-4-NP**. (D and E) Fluorescence images and results of quantitative analysis of tumor samples at 0, 1, 2, and 3 min after spraying. (F) Schematic illustration of **S-4-NP** applied in a radical nephrectomy. (G) Representative pure fluorescence (left) and pseudocolor (right) images of a fully transected whole tumorous kidney sprayed with **S-4-NP**. **P < 0.01. NIR, near-infrared. (H) Graphic summary.

The clinical NIR tumor imaging efficacy of compound **4** (**S-4-NP**)-based nanoparticles was evaluated in a NSS (*n* = 7). The technical process is shown in Fig. 2A. After the tumor was removed, the surgeon separated a 3–5 mm thick section from the excised tissue using a scalpel. Then, the **S-4-NP** solution was sprayed onto the section. Optical imaging was performed at 1, 2, and 3 min after spraying with an intraoperative NIR imaging device. The representative pseudocolor image (an NSM specimen) is shown in Fig. 2B. The cancerous tissue under image became distinguishable from non-cancerous tissues in less than 1 mm. The boundary between the tumor and the capsule could be clearly identified; this was further supported by the results of hematoxylin and eosin staining analysis (Fig. 2C). The results of the quantitative analysis showed that, compared to that in the capsule and normal kidney tissue, the NIR fluorescence signal in the tumor was significantly increased by approximately 4-fold (Fig. 2D). The fluorescence images taken at 0 min (before the test), 1, 2, and 3 min are shown in Fig. 2E. The first clear imaging result was obtained in 1 min, and the fluorescence signal increased over time. The full video recording of the imaging process is provided in the supplementary information.

In addition, to obtain the upper limit of the field of vision in our method, we investigated the imaging properties of **S-4-NP** during radical nephrectomy (*n* = 3) for the purpose of academic research (Fig. 2F). A full section of the whole kidney containing the tumor was prepared (Fig. 2G) and the field of vision could reach up to 10 cm × 10 cm.

Furthermore, the imaging properties of compound **1**, **2**, **3**, **5**, and **6** were also evaluated using clinical samples (Fig. S8), with compound **1**, **2**, and **3** being evaluated using normal kidney tissues and compound 5 and 6 being evaluated using cancerous tissues. A strong NIR signal was observed in the normal tissue at 1 min using compound **1**, **2**, and **3**, indicating that they lacked tumor-specific targeting properties. For compound **5** and **6**, the image appeared slower than compound 4, and the fluorescence signal in the tumor tissue was weak. These results indicated that the stability of the nanoparticles plays a key role in the imagery: (i) unstable nanostructures could not achieve tumor targeted imaging, due to serious nonspecific staining, for example, compound **1**, **2**, and **3**; (ii) unduly stable nanostructures affected the cellular uptake rate of the dye (the uptake value of compound **5** and **6** was much lower than that of compound **4**). Thus, the **S-4-NP** nanoparticles was the optimal formula in this study.

This work reported a simple, effective, and well-designed approach to tumor tissue imaging. It could be widely applicable to cancer operations and provide obvious advantage over existing surgical methods. Our research group and partner organizations have begun further clinical evaluations of **S-4-NP** and its derivatives in other tumor types, such as breast, bladder, lung, colorectal, prostate, and brain cancers.

## Research limitations

As a new release mode, the mechanism of the active release mode requires further and deeper understanding. Due to technical limitations, our research in this report only provided qualitative evidences but little direct or quantitative measurement. Future mechanistic insights are needed and relevant work is under way.

## Supplementary Material

lnac059_suppl_Supplementary_Material

lnac059_suppl_Supplementary_Video
