# Supplementary material for: A novel spraying nanoprobe for renal cell carcinoma in humans
Source: Life Med. 2022 Dec 23;2(1):lnac059. doi: 10.1093/lifemedi/lnac059 (PMC11749079; doi:10.1093/lifemedi/lnac059)
Supplement: lnac059_suppl_Supplementary_Material [file lnac059_suppl_Supplementary_Material.docx]

**Supporting Information**

**A Novel Spraying Nanoprobe for Renal Cell Carcinoma in Humans**

**1. Chemistry**

**Synthesis of compounds 1-7**

All solvents were purified and dried by standard procedures and kept over a suitable drying agent prior to use. Unless otherwise noted, all reagents were purchased from commercial sources and were used without further purification. The reactions were monitored by thin layer chromatography coated with silica gel.

^1^H NMR and ^13^C NMR data were acquired in CDCl_3_ as solvent on a Bruker DMX 500 spectrometer. The chemical shifts (*δ*) are expressed in ppm (parts per million) relative to TMS (tetramethylsilane). Spin-spin coupling constants (*J*) were measured directly from the spectra and were given in Hz. Exact mass (HR-MS) spectra were recorded on Thermo Fisher Scientific LTQ FT Ultra.

Compound **1-3,8** was synthesized and purified as reported (*ACS Appl. Bio Mater.* **2020**, 3, 327−334).

Synthesis of compounds **4**: Briefly, to a stirred and cooled (0°C) solution of the compound **3** (68 mg, 0.1 mmol) and phenol (18.8 mg, 0.2 mmol) in CH_2_Cl_2_ (2 mL), the solution of EDC-HCl (23 mg, 0.12 mmol) and DMAP (5 mg, 0.04 mmol) in CH_2_Cl_2_ (4 mL) was slowly added over 0.5 h. After stirring at RT for 10 h, CH_2_Cl_2_ (20 mL) was added. The solution was washed with saturated 1N HCl (20 mL), brine (20 mL) and then dried over Na_2_SO_4_. The solvent was removed to give the crude product, which was subsequently purified by silica gel column chromatography (10% v/v methanol/CH_2_Cl_2_) to afford the pure compounds **4** as a green solid (65 mg, 86 % yield).

Characterization of compounds **4**: ^1^H NMR (500 MHz, CD_3_Cl_,_ *δ*): 8.37-8.31 (m, 2H), 7.54-7.53 (m, 1H), 7.41-7.35 (m, 7H), 7.25-7.12 (m, 4H), 7.03-7.01 (m, 1H), 6.32-6.31 (m, 1H), 6.30-6.29 (m, 1H), 4.32-4.26 (m, 4H), 2.76-2.73 (m, 4H), 2.63-2.60 (m, 2H), 1.96-1.83 (m, 8H), 1.72-1.71 (m, 12H), 1.47 (t, *J* = 7.0 Hz, 3H). ^13^C NMR (125 MHz, CD_3_Cl_,_ *δ*): 172.1, 171.9, 150.6, 150.2, 144.5, 143.9, 129.4, 128.9, 127.6, 125.8, 125.3, 122.2, 121.5, 110.8, 101.5, 49.4, 49.2, 44.6, 39.9, 33.9, 31.9, 31.4, 30.2, 29.7, 29.4, 28.2, 28.0, 27.2, 26.6, 26.4, 24.5, 22.7, 20.7, 14.1, 12.5. HRMS: *m/z* [M]^+^ calc for C_44_H_50_ClN_2_O_2_^+^: 673.3555; found: 673.3550.

Synthesis of compounds **5**: Briefly, to a stirred and cooled (0°C) solution of the compound **3** (68 mg, 0.1 mmol) and *tert*-butyl 4-hydroxyphenylcarbamate (42 mg, 0.2 mmol) in CH_2_Cl_2_ (2 mL), the solution of EDC-HCl (23 mg, 0.12 mmol) and DMAP (5 mg, 0.04 mmol) in CH_2_Cl_2_ (4 mL) was slowly added over 0.5 h. After stirring at RT for 10 h, CH_2_Cl_2_ (20 mL) was added. The solution was washed with saturated 1N HCl (20 mL), brine (20 mL) and then dried over Na_2_SO_4_. The solvent was removed to give the crude product, which was subsequently purified by silica gel column chromatography (10% v/v methanol/CH_2_Cl_2_) to afford the pure compounds **4** as a green solid (77 mg, 89 % yield).

Characterization of compounds **5**: ^1^H NMR (500 MHz, CD_3_Cl_,_ *δ*): 8.38-8.29 (m, 2H), 7.43-7.37 (m, 7H), 7.25-7.21 (m, 2H), 7.15-7.14 (m, 1H), 6.95-6.93 (m, 2H), 6.38-6.35 (m, 1H), 6.26-6.23 (m, 1H), 4.36-4.32 (m, 2H), 4.24-4.21 (m, 2H), 2.76-2.70 (m, 4H), 2.60-2.57 (m, 2H), 1.93-1.85 (m, 8H), 1.72-1.71 (m, 12H), 1.51 (s, 9H), 1.47 (t, *J* = 7.0 Hz, 3H). ^13^C NMR (125 MHz, CD_3_Cl_,_ *δ*): 172.4, 172.0, 171.6, 152.9, 150.2, 145.8, 144.8, 143.6, 136.4, 128.9, 128.0, 127.6, 125.6, 125.0, 122.2, 121.7, 110.8, 110.5, 101.9, 101.1, 49.5, 49.1, 44.4, 40.0, 33.8, 31.2, 29.7, 28.3, 28.1, 28.0, 27.1, 26.6, 26.3, 24.5, 22.7, 20.7, 14.4, 12.5. HRMS: *m/z* [M]^+^ calc for C_49_H_59_ClN_3_O_4_^+^: 788.4189; found: 788.4185.

Synthesis of compounds **6**: Briefly, to a stirred and cooled (0°C) solution of the compound **3** (68 mg, 0.1 mmol) and 1-adamantaneethanol (36 mg, 0.2 mmol) in CH_2_Cl_2_ (2 mL), the solution of EDC-HCl (23 mg, 0.12 mmol) and DMAP (5 mg, 0.04 mmol) in CH_2_Cl_2_ (4 mL) was slowly added over 0.5 h. After stirring at RT for 10 h, CH_2_Cl_2_ (20 mL) was added. The solution was washed with saturated 1N HCl (20 mL), brine (20 mL) and then dried over Na_2_SO_4_. The solvent was removed to give the crude product, which was subsequently purified by silica gel column chromatography (10% v/v methanol/CH_2_Cl_2_) to afford the pure compounds **6** as a green solid (68 mg, 81 % yield).

Characterization of compounds **6**: ^1^H NMR (500 MHz, CD_3_Cl_,_ *δ*): 8.38-8.30 (m, 2H), 7.54-7.52 (m, 1H), 7.44-7.35 (m, 4H), 7.25-7.22 (m, 2H), 7.15-7.13 (m, 1H), 6.35-6.33 (m, 1H), 6.26-6.23 (m, 1H), 4.36-4.31 (m, 2H), 4.22-4.19 (m, 2H), 4.10 (t, *J* = 7.5 Hz, 2H), 2.78-2.73 (m, 4H), 2.34-2.31 (m, 2H), 2.03-1.83 (m, 8H), 1.72-1.71 (m, 12H), 1.68-1.60 (m, 7H), 1.51-1.50 (m, 6H), 1.47 (t, *J* = 7.0 Hz, 3H), 1.40-1.33 (m, 4H). ^13^C NMR (125 MHz, CD_3_Cl_,_ *δ*): 173.6, 172.3, 171.7, 150.7, 144.8, 143.7, 142.3, 128.9, 128.7, 125.5, 125.1, 122.2, 110.9, 110.6, 101.7, 101.5, 60.9, 49.4, 49.1, 44.5, 42.4, 42.3, 40.0, 36.9, 34.8, 34.0, 31.9, 31.7, 31.4, 30.2, 29.7, 29.5, 29.3, 29.1, 28.5, 28.1, 28.0, 27.1, 26.6, 26.4, 24.6, 22.7, 20.7, 14.1, 12.5. HRMS: *m/z* [M]^+^ calc for C_50_H_64_ClN_2_O_2_^+^: 759.4651; found: 759.4648.

Synthesis of compounds **7**: Briefly, as showed in Scheme **S1,** to a stirred and cooled (0 °C) solution of the compound **8** (57 mg, 0.1 mmol) and phenol (18.8 mg, 0.2 mmol) in CH_2_Cl_2_ (2 mL), the solution of EDC-HCl (23 mg, 0.12 mmol) and DMAP (5 mg, 0.04 mmol) in CH_2_Cl_2_ (4 mL) was slowly added over 0.5 h. After stirring at RT for 10 h, CH_2_Cl_2_ (20 mL) was added. The solution was washed with saturated 1N HCl (20 mL), brine (20 mL) and then dried over Na_2_SO_4_. The solvent was removed to generate the crude product, which was subsequently purified by silica gel column chromatography (10% v/v methanol/CH_2_Cl_2_) to obtain the pure compounds **7** as a blue solid (59 mg, 91% yield).

Characterization of compounds **7**: ^1^H NMR (500 MHz, CD_3_Cl_,_ *δ*): ^1^H NMR (500 MHz, CD_3_Cl_,_ *δ*): 8.20 (t, *J* = 13.0 Hz, 2H), 7.38-7.35 (m, 6H), 7.24-7.20 (m, 3H), 7.08 (t, *J* = 8.0 Hz, 2H), 7.05-7.03 (m, 2H), 6.85 (t, *J* = 12.5 Hz, 2H), 6.45 (d, *J* = 13.5 Hz, 1H), 6.38 (d, *J* = 13.5 Hz, 1H), 4.16-4.11 (m, 5H), 2.62 (t, *J* = 7.5 Hz, 2H), 1.88-1.82 (m, 4H), 1.76 (s, 12H), 1.42 (t, *J* = 7.0 Hz, 3H). ^13^C NMR (125 MHz, CD_3_Cl_,_ *δ*): 154.2, 154.1, 150.6, 142.1, 141.7, 141.5, 141.3, 129.4, 128.6, 126.7, 125.8, 125.0, 124.9, 122.4, 121.6, 110.3, 110.2, 103.9, 103.7, 49.4, 49.3, 44.2, 39.4, 33.9, 29.7, 28.1, 27.9, 27.2, 26.3, 24.5, 12.5. HRMS: *m/z* [M]^+^ calc for C_39_H_45_N_2_O_2_^+^: 573.3476; found: 573.3469.

**2. Preparation and characterization of dyes with Surfactin**

In brief, dyes were rapidly dissolved in Surfactin solution, and the solution was vigorous stirred at 25°C for 5 min. The mixture filtrated through 0.22 μm filter membrane to remove the unincorporated dyes aggregates.

The NIR imaging was taken using Xenogen IVIS Spectrum imaging system.

The fluorescence spectra were determined by Angilent Cary Elipse fluorescence spectrophotometer.

The size and distributions of the particles were measured by a dynamic light scattering (DLS) spectrometer using a Zetasizer Nano ZS instrument (Malvern, Worcestershire, UK).

The morphological examination of particles was performed by 120 kV JEOL JEM-1400 Plus biology transmission electron microscopy with a negative stain method (1% phosphotungstic acid).

**3. Cell culture**

Human 786-0 cell lines was provided by Shanghai Ninth People’s Hospital. 786-0 cells were maintained in RPMI-1640 medium (HyClone), and supplemented with 10% fetal bovine serum (FBS, Gibco BRL, USA) in a 5% CO_2_/95% air humidified atmosphere at 37°C.

**4. Fluorescence microscopy assay**

786-0 cells (3 × 10^5^ per well) were seeded on microscope cover glass (Fisherbrand) in a six-well plate and were allowed to form a monolayer over 48 h. Then cells were exposed to dyes (2 µM) and Mito-tracker Green (200 nM) in 500 µL of fresh medium with 10% FBS. The glasses were incubated at 37°C for 0.5 h, and washed three times with PBS to remove excess dyes. Then cells were fixed with 4% formaldehyde at room [temperature](javascript:void(0);) for 15 minutes. The glasses were then washed three times with PBS. Images were recorded by fluorescent microscopy analysis (IX83, Olympus, Japan) using a LED illuminant (SpectraX, Lumencor, Germany) infrared camera (Prime, Photometrics, USA) with excitation at 460–520 nm (for Mito-tracker Green) and emission at 810–890 nm (for dyes) wavelength.

**5. Mouse xenograft model**

This study was performed in strict accordance with the NIH guidelines for the care and use of laboratory animals (NIH Publication No. 85-23 Rev. 1985), and all animal protocols were approved by the Institutional Animal Care and Use Committee of Shanghai Jiao-Tong University School of Medicine (SJTUSM IACUC).

Five-week-old female nude mice were purchased from Shanghai Slack Laboratory Animal Co., LTD, and maintained in a standard environment. The mice were allowed to acclimatize for at least 1 week prior to the experiment. For subcutaneous xenograft studies, 786-0 cells (3.0×10^6^ per mouse) suspended in 0.1 mL of PBS were inoculated subcutaneously on the right flank of each mouse. Tumor size was measured by caliper.

**6. NIR imaging in animal models**

For mice bearing xenografted tumors, when tumor sizes reached 100-150 mm^3^, tumors were dissected and the test solution was immediately sprayed onto the cancerous tissue. NIR imaging was performed at 3, 6, 9 and 12 min. NIR imaging were taken using Xenogen IVIS Spectrum imaging system (compounds **1**-**6**, excitation/emission, 745/820 nm; compounds **7**, excitation/emission, 640/680 nm).

**7. Verification of the active release mode**

Most previously designed nanoparticles enter the target cell as a complete structure, releasing the loaded drug intracellularly. In contrast with these traditional drug delivery systems, our results indicated that our system featured an active release mode: only loaded dyes entered the targeted cell, rather than the entire nanoparticle. To verify this unconventional release mode, we prepared a contrast dye, compound **7**; its structure is shown in **Fig. S6A** Compared to compound **4**, compound **7** lacked the rigid 1-chlorocyclohexenyl substitution in the polymethine linker. Therefore, compound **7** could not specifically identify the cancer cell with OATPs (**Fig. S7**). In addition, the maximum emission wavelengths of compound **4** and compound **7** differed (820 nm for compound **4**; 680 nm for compound **7**).

A three-component nanoparticle (**S-4-7-NP**) was subsequently synthesized that contained compound **4**, compound **7**, and surfactin in a molar ratio of 0.5:0.5:5. First, the DLS data indicated that **S-4-7-NP** was about 85 nm in size, with good polydispersity (DPI < 0.2) (Table **S1**). Second, the fluorescence properties of **S-4-7-NP** indicated that the fluorescence of both compounds **4** and **7** was quenched in the particles (**Fig.**  **S6B-D**). Finally, to verify the presence of an active release mode, we evaluated the uptake of **S-4-7-NP** in cancerous tissues from the mouse model. If **S-4-7-NP** entered the cells as whole particles via phagocytosis, compounds **4** and **7** would enter the cells together, and the signal intensities of the two dyes would exhibit the same trend. However, our imaging and quantitative analysis results showed that the range of uptake values increased by 40% and 135% for compounds **7** and **4**, respectively, from 3 min to 12 min (**Fig.**  **S6E-F**). This indicated that most of compound **4** was released from the nanoparticle structure and independently entered the cells via OATP recognition. The schematic diagram of the **S-4-7-NP** release mode is illustrated in **Fig.**  **S6G**.

**8. NIR imaging in** **clinical trials**

This study has been approved by the ethics committee of Shanghai Ninth People’s Hospital (Shanghai, China).

The entire process was completed under the supervision of professional pathologist.

The tissue was removed from patient. The surgeon determined detection location of excised tissue and cut into piece （3–5 mm thick）by scalpel. The test solution was sprayed onto the section. The optical imaging is gained with an intraoperative NIR imaging device (Nuoyuan, Nanjing, China).

**9. Statistical analysis**

Results of quantitative measurements were expressed as mean ± standard deviation (SD). All statistical analyses were performed with the SPSS software package (version 17.0, SPSS, Inc., Chicago, IL).


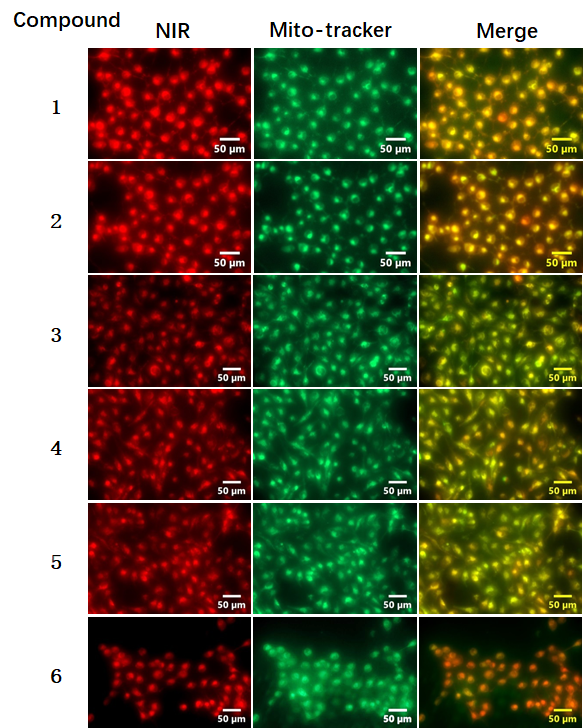


**Figure S1**. Representative fluorescent images for dyes in 786-0 cells by fluorescent microscopy analysis. Mito-tracker Green was used as mitochondria-specific dye.

**
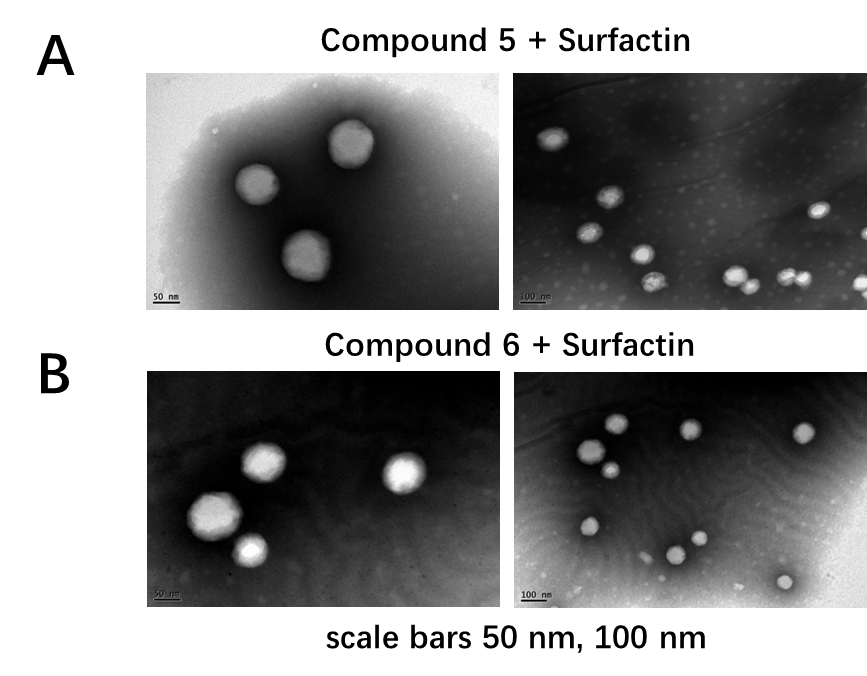
**

**Figure S2.** Transmission electron microscopy images of dyes combined with surfactin in a 1:5 molar ratio: (A) compound **5**, (B) compound **6**. Scale bars: 50 nm, 100 nm. NIR, near-infrared.

**
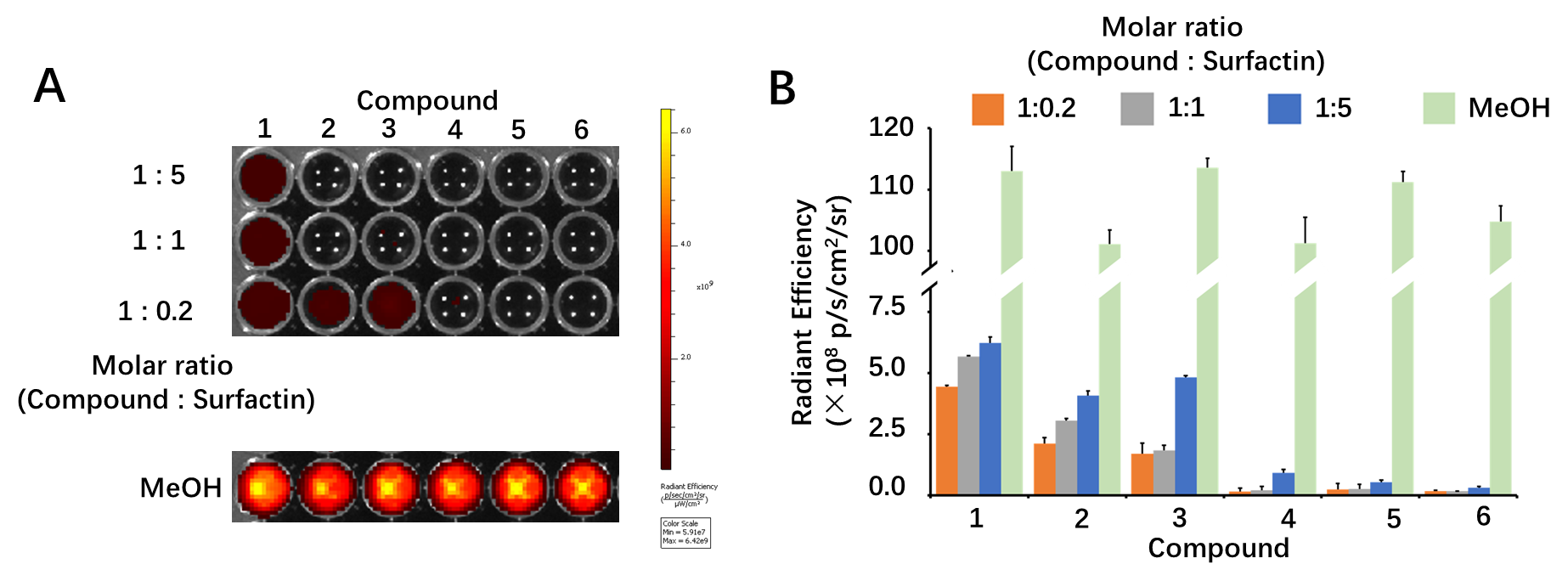
**

**Figure S3.** (A) Representative fluorescence images of dyes combined with surfactin. (B) Quantitative analysis of the fluorescence images.


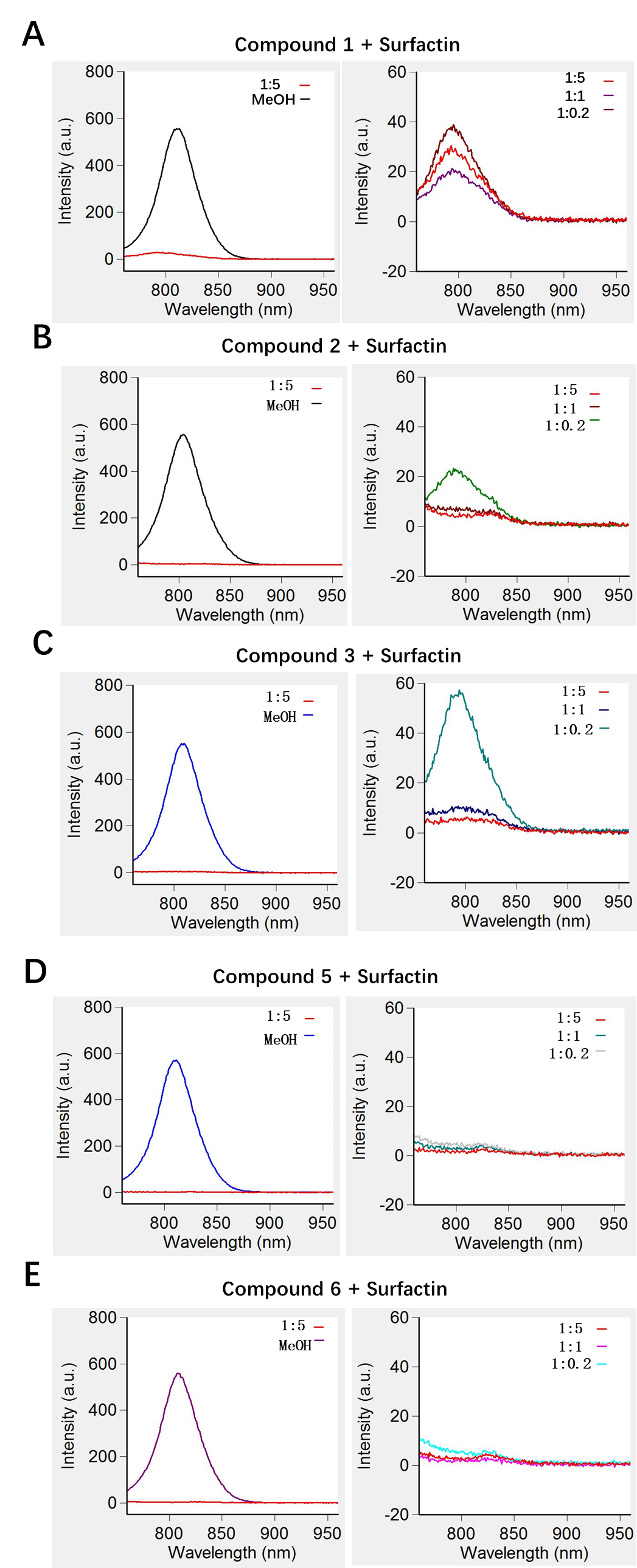


**Figure S4.** The fluorescence spectra of dyes with Surfactin (excitation at 730 nm and the emission from 750 to 960 nm): (A) for compound **1**, (B) for compound **2**, (C) for compound **3**, (D) for compound **5**, (E) for compound **6**.


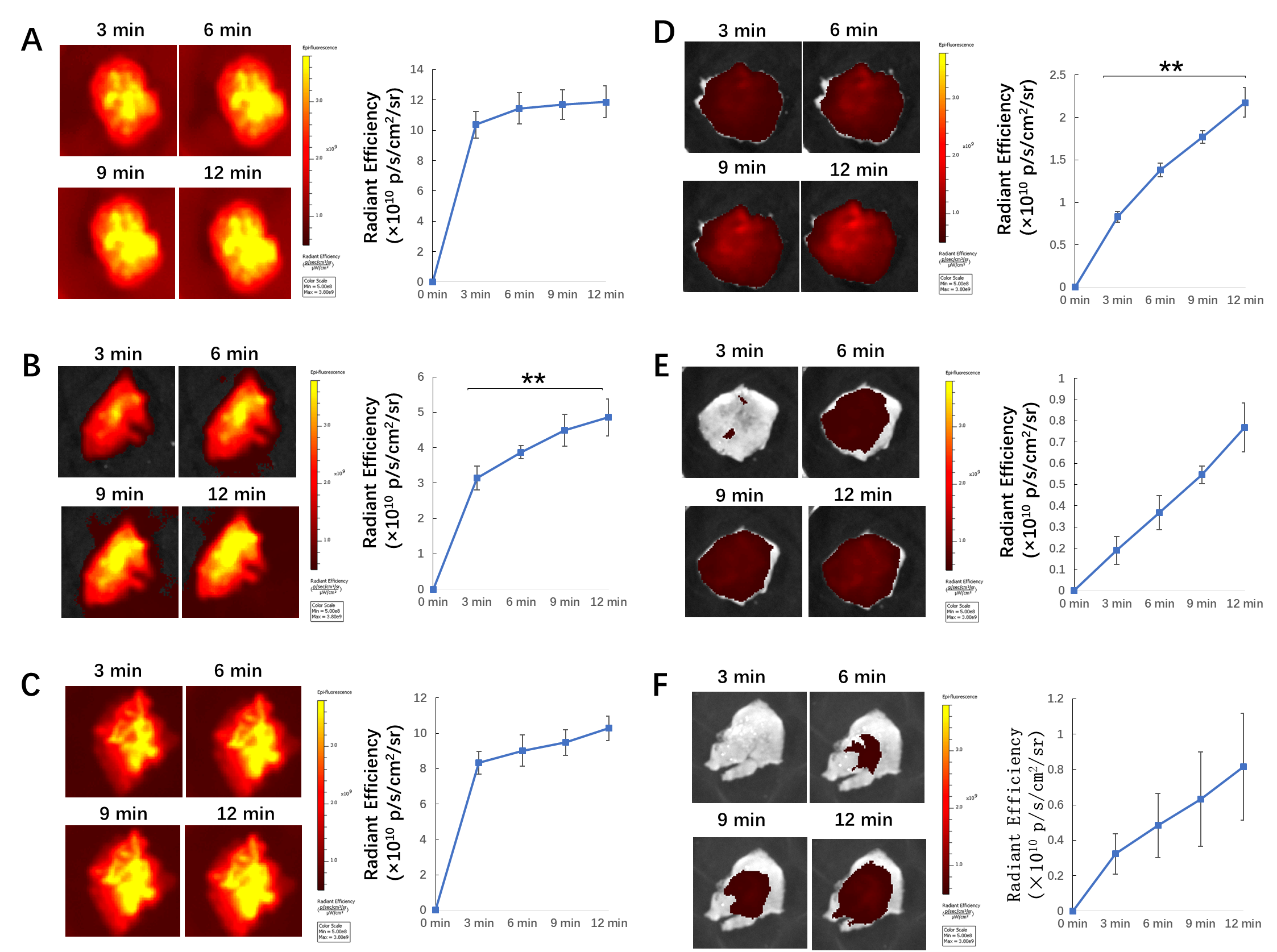


**Figure S5.** NIR imaging study of 786-0 tumor-bearing mice (*n* = 3) (representative fluorescence images, left; quantitative analysis results, right): (A) compound **1**, (B) compound **2**, (C) compound **3**, (D) compound **5**, (E) compound **6**, and (F) compound **4** with bromosulfophthalein (50 times eq.). ***p* < 0.01.


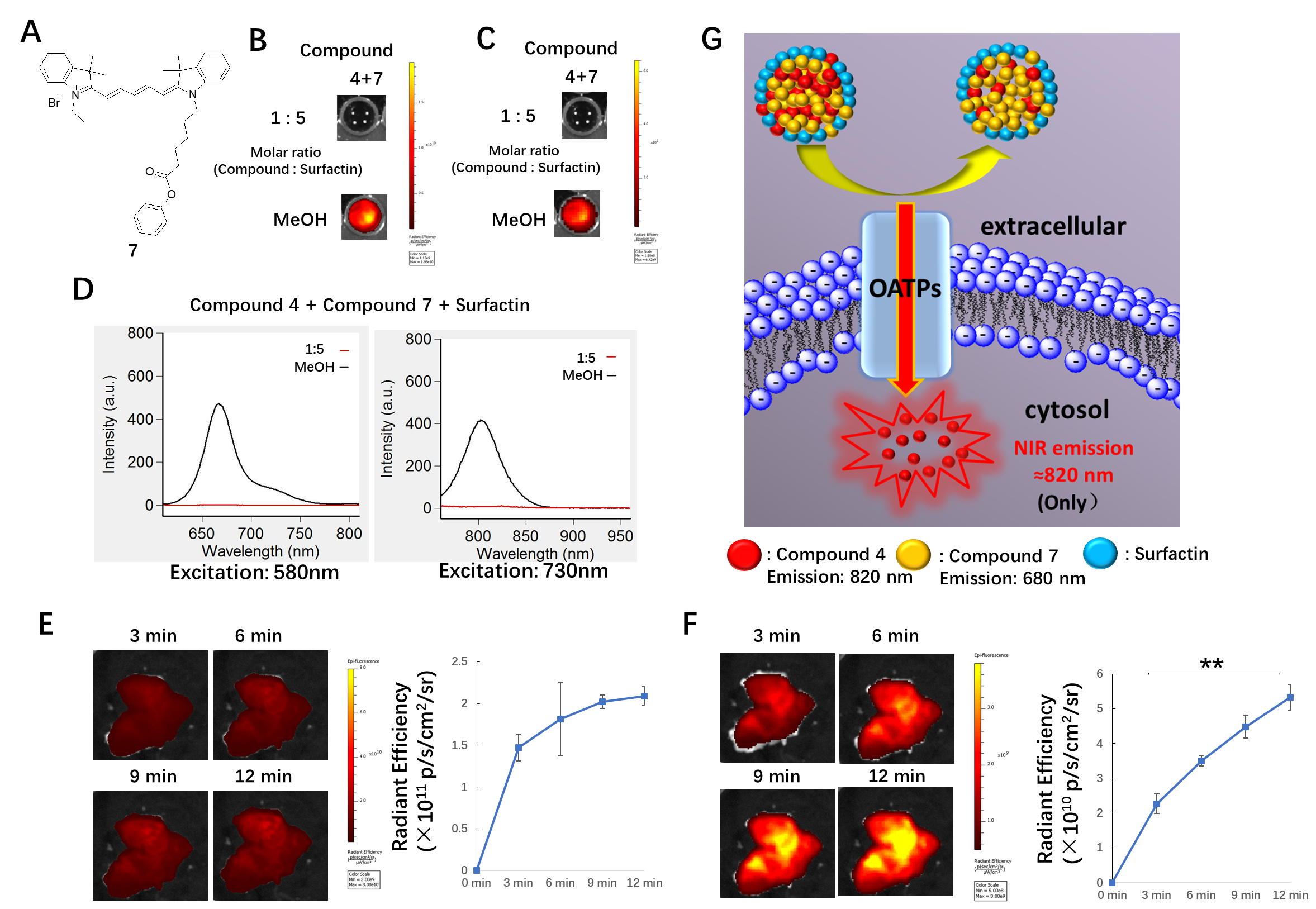


**Figure S6.** (A) The structure of compound **7**. Representative fluorescence images of **S-4-7-NP**: (B) compound **7** signal (excitation/emission, 640/680 nm); (C) compound **4** signal (excitation/emission, 745/820 nm). (D) The fluorescence spectra of **S-4-7-NP** (excitation at 580 nm and emission from 610 to 810 nm for compound **7**; excitation at 730 nm and emission from 750 to 960 nm for compound **4**). NIR imaging study of **S-4-7-NP** (*n* = 3): (E) compound **7** signal (excitation/emission, 640/680 nm); (F) compound **4** signal (excitation/emission, 745/820 nm). (G) A schematic illustration showing the cell uptake mode of **S-4-7-NP**. ***p* < 0.01. OATPs, organic anion-transporting polypeptides; NIR, near-infrared.


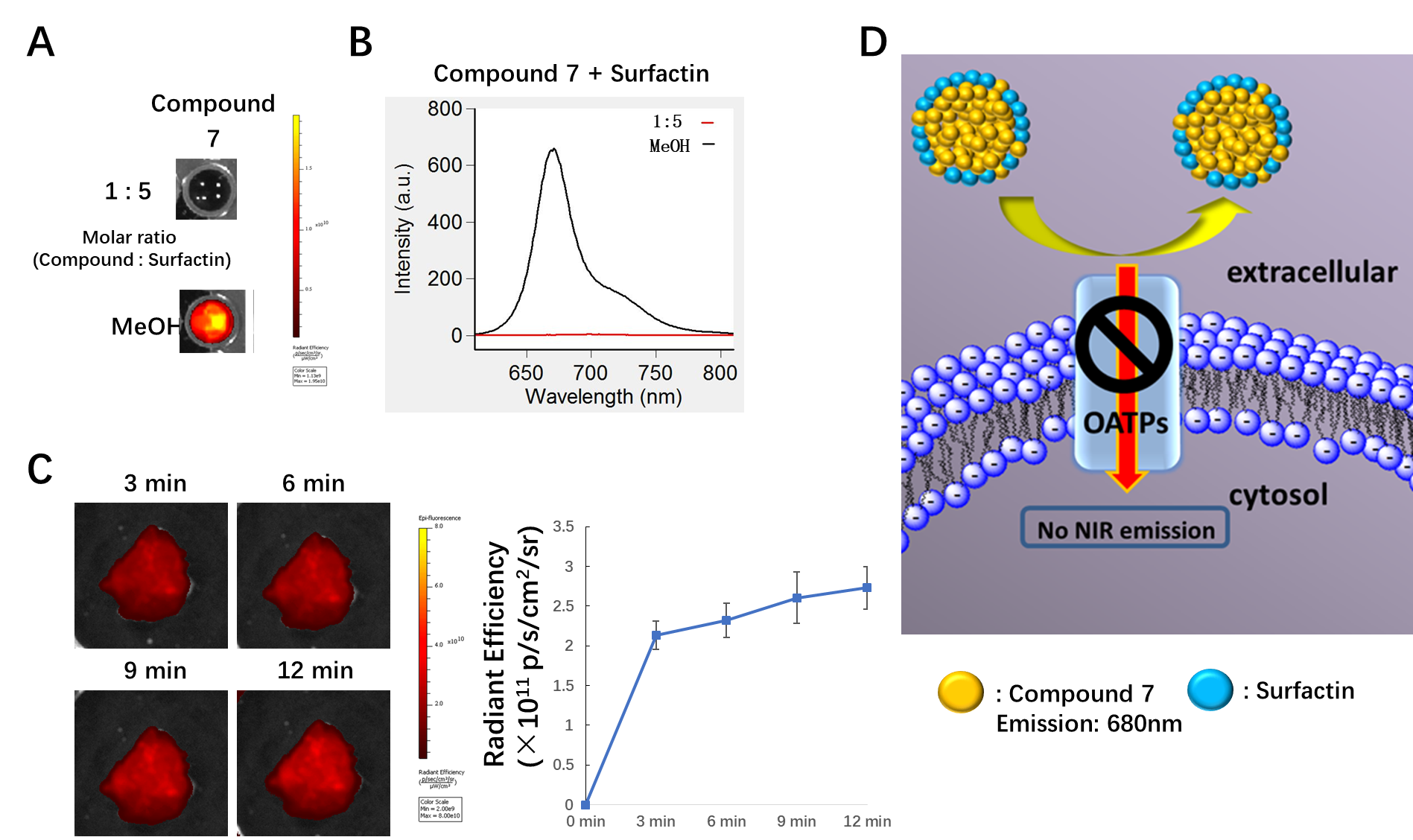


**Figure S7.** Study of compound **7** with Surfactin (**S-7-NP**). (A) Representative fluorescent images of **S-7-NP**: for compound **7** signal (excitation/emission, 640/680 nm) (B) The fluorescence spectra of **S-7-NP** (excitation at 580 nm and the emission from 610 to 810 nm). (C) NIR imaging study of **S-7-NP** (*n* = 3) (excitation/emission, 640/680 nm) using 786-0 xenograft mouse models. (D) A schematic illustration showing the cell uptake mode of **S-7-NP**.


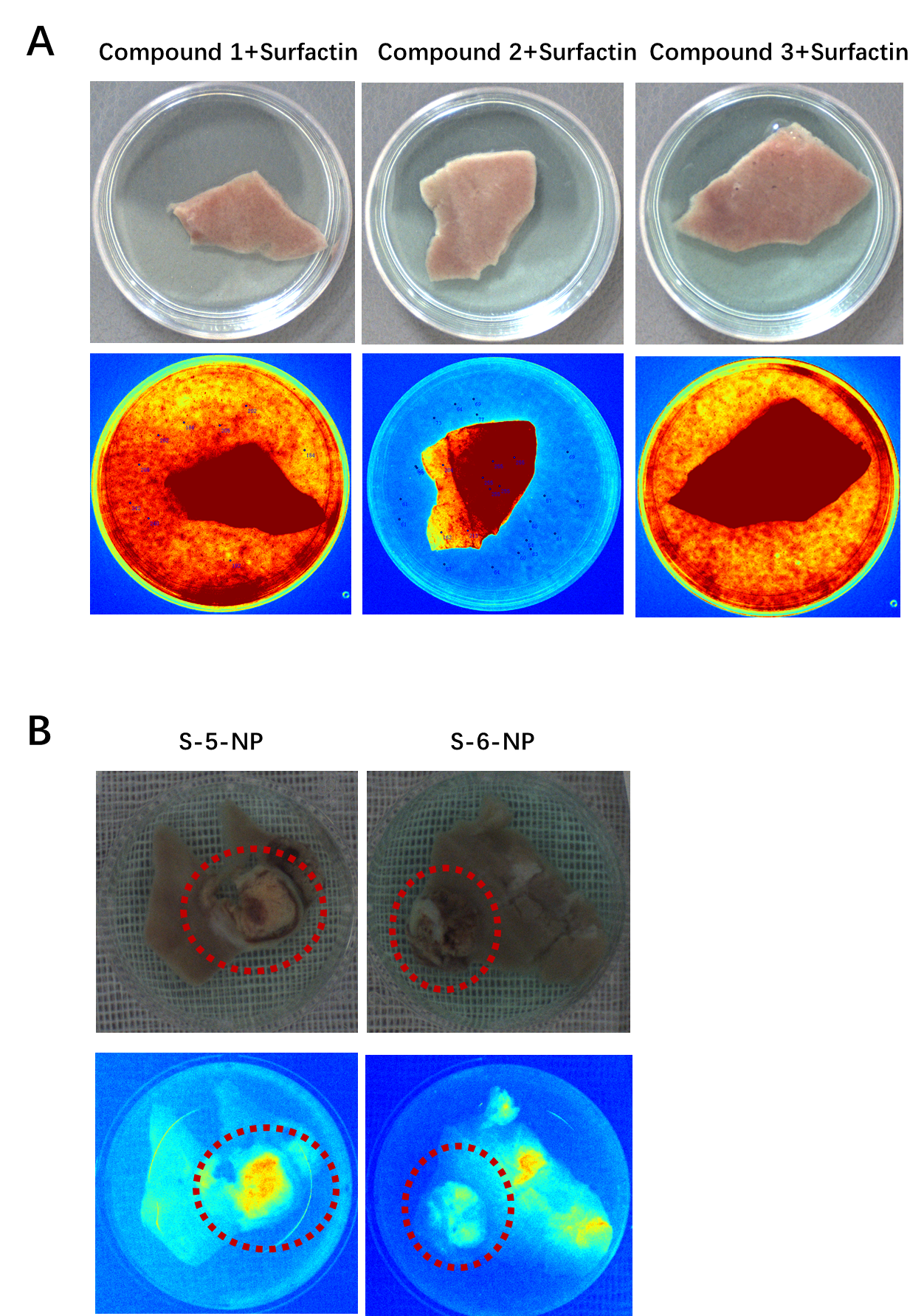


**Figure S8.** (A) Representative pseudocolor image of transection section of normal kidney tissue for dyes and Surfactin in 1 minute. (B) Representative pseudocolor image of transection section of tumor tissue for dyes and Surfactin in 6 minutes (red circle indicates the tumor).


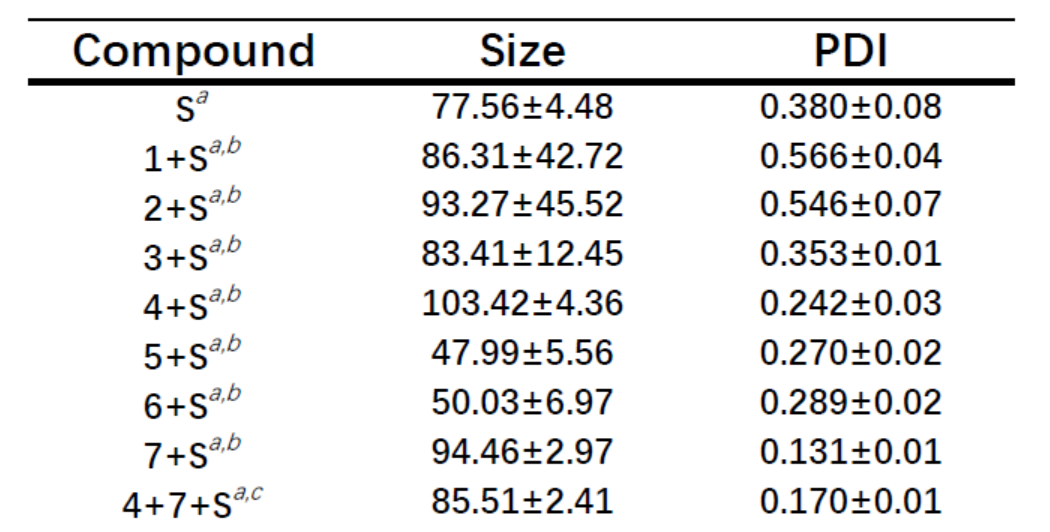


**Table S1**. Characterization of dyes with Surfactin as determined by dynamic light scattering (DLS). *^a^* The concentration of Surfactin was 100 μM. *^b^* The molar ratio was 1:5 (compound *vs.* Surfactin). *^c^* The molar ratio was 0.5:0.5:5 (compound 4 *vs.* compound 7 *vs.* Surfactin).


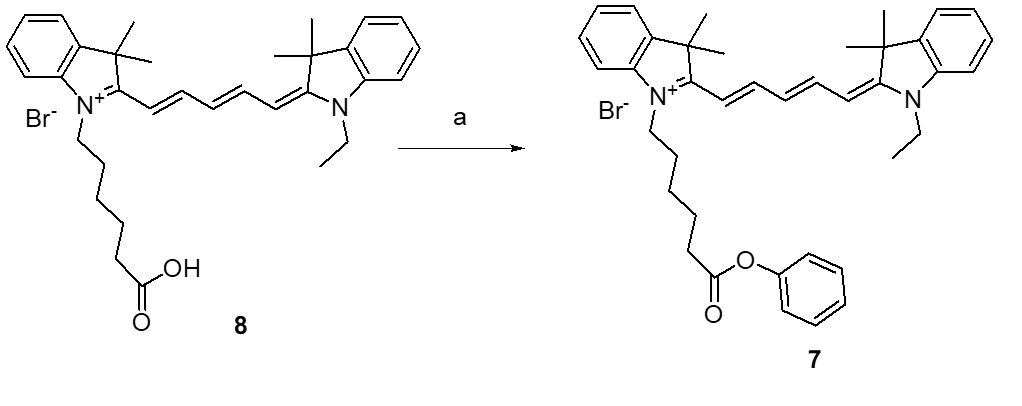


**Scheme S1.** Synthesis of Er-Cy5. Reagents: (a) phenol, EDC, DMAP, CH_2_Cl_2_.
